# Drugs for malaria elimination: what do we have now and what do we need?

**DOI:** 10.1186/1475-2875-13-S1-O35

**Published:** 2014-09-22

**Authors:** Dennis Shanks

**Affiliations:** 1 Army Malaria Institute & The University of Queensland, Australia

## 

One has to use antimalarial drugs differently for elimination than ordinary treatment. When treating sick patients, the objective is to kill many parasites in a few sick people whereas malaria elimination involves killing a few parasites in many well people. There are standard public health means to reach very low levels of malaria infection; what is difficult is moving from low to no malaria. Although mass drug administration has been used successfully in China, it is not entirely clear how such methods might be adapted for other populations especially those with more persons having G6PD deficiency. Primaquine and methylene blue are among our oldest antimalarial drugs and are established means of killing the gametocyte transmission stages. Adverse events and long dosage regimens make both primaquine and methylene blue far from the mass drug administration model used during antifilarial drug campaigns. A long acting primaquine analog, tafenoquine, is in late clinical trials and may have some role in eliminating relapsing malaria hypnozoites from a population. Other new chemical compounds such as the imidazole-piperazine KA156 are in the development pipeline which have anti-transmission potential although this has not be demonstrated in field trials to date. Eliminating malaria will require a multi-faceted approach that will necessarily involve new means of using drugs to kill small residual parasite populations long after the major public health effects of malarial disease has disappeared.

